# Implementation of the Registered Nurses’ Association of Ontario Best Practice Guidelines for delirium-specific recommendations in a digital practice setting at Humber River Health, Canada

**DOI:** 10.1016/j.ijnss.2026.04.015

**Published:** 2026-04-25

**Authors:** Jennifer Yoon, Derek Hutchinson, Amanpreet Ghuman, Gurmit Maghera, Beatrise Edelstein, Rachelle Soogree, Maritess Payumo, Grace Mercieca, Shirley Goguen, Elizabeth Leblanc, Akhil Plathanathu, Abhimanyu Sud, Barbara E. Collins, Teodora Neagu, Aleksandra Zuk

**Affiliations:** a Hennick Humber Hospital Site, Humber River Health, Toronto, Canada; b School of Nursing, Queen’s University, Kingston, Canada; c Laurence Bloomberg Nursing, University of Toronto, Toronto, Canada; d Department of Family and Community Medicine, University of Toronto, Toronto, Canada

**Keywords:** Best practice guidelines, Delirium, Electronic health records, Evidence-based practice, Nursing, Patient safety

## Abstract

**Objective:**

This study aimed to outline and evaluate three key implementation strategies undertaken by Humber River Health (HRH) nursing leadership to support the sustained implementation of the delirium, dementia & depression Best Practice Guideline (BPG).

**Methods:**

In 2017, HRH embarked on a high-reliability journey, prioritizing consistent quality and safe care delivery by implementing the Registered Nurses’ Association of Ontario (RNAO) Best Practice Guidelines (BPGs). Based on the implementation guidelines, the hospital has also adopted three measures: the use of electronic medical records “DocOpt”, embedding the content of delirium into staff training, and constructing health risk blocks with hierarchical early warnings, embedded within our Command Center. From 2019 to 2025, a retrospective longitudinal assessment of hospital inpatients was conducted using process indicators, outcome indicators, and patient satisfaction. Process and outcome metrics were evaluated pre- and post-implementation using Statistical Process Control Charts in Microsoft Excel QI Macros, in which Central Lines and Upper and Lower Lines were calculated. Further, segmented regression was conducted to evaluate key time periods during the implementation of the delirium BPG.

**Results:**

Due to the implementation of specific recommendations for delirium, the process indicators of delirium in HRH patients improved from 2019 to 2025, and the incidence of delirium in elderly patients per 1,000 patient care days decreased accordingly, by 1.4 times (accounting for 23.3 %); more than 90 % of patients and their families were satisfied with the hospital. According to the segmented regression analysis, it indicates that from the third quarter of 2019 to the third quarter of 2020, and from the first quarter of 2021 to the first quarter of 2022, there was a transitional period, which marked the period when the “DocOpt” system and health risk blocks were applied.

**Conclusions:**

Long-term evaluation of process and outcomes data supported HRH to achieve improved patient delirium outcomes. Future research can rely on the regular, dynamic data-monitoring system to provide more empirical evidence for the localization adaptation, consistent implementation, and continuous quality improvement of BPGs.

## What is known?

• Implementing evidence-based practice improves patient outcomes.
• Implementing strategies at multiple levels within an organization improves potential for sustained practice.

## What is new?

• Sequencing, scaffolding, and integrating strategies can set organizations up for long-term success.
• Hospital electronic medical records should integrate the Registered Nurses’ Association (RNAO) Best Practice Guidelines (BPGs).
• Both short-term (through real-time responses) and long-term evaluation are required to ensure adherence to the RNAO BPGs and identify additional opportunities for practice improvement.

## 1 Introduction

Delirium in older adults is a common neuropsychiatric syndrome marked by a sudden change in a person’s mental function, typically lasting one to seven days [1]. The Registered Nurses’ Association of Ontario (RNAO) states that up to 50 % of hospitalized older patients are affected by delirium, creating substantial system burden [1,2]. In 2016, RNAO sought to address this clinical issue of delirium in a healthcare setting by publishing a Best Practice Guideline (BPG) to enhance the quality of practice for delirium, dementia, and depression. The document details recommendations for care and assessment through practice, education, and organization/policy.

Beginning in 2016/17, Humber River Health (HRH) embarked on a journey to become a high-reliability hospital and sought to achieve a Best Practice Spotlight Organization® (BPSO®) designation by the RNAO. BPSO® designation requires meeting numerous specific requirements, which include: systematic implementation of various BPGs, establishment of supporting infrastructures for best clinical practice, providing reports of BPG implementation, participating in various knowledge exchange forums, and ensuring sustained implementation and evaluation of standardized indicators through a dedicated RNAO platform called Nursing Quality Indicators for Reporting and Evaluation (NQuire).

The HRH nursing executive leadership examined the hospital’s performance in the Canadian Institute for Health Information (CIHI) Hospital Harm project. CIHI reported HRH’s patient-harm rate as 3.4 % (the crude rate of patient harm divided by patient discharges) in 2016/17. A closer examination revealed that one of the highest reported clinical incidents year over year was delirium. Thus, delirium management was selected as a clinical priority to implement in the BPSO® program.

Implementing major organizational changes at HRH required multiple strategies to improve patient outcomes [3,4]. Despite a global commitment to adopting BPGs, systematic reviews have identified that implementation strategies in nursing, in particular, are complex and often require multi-level, multifaceted approaches such as education, local adaptation, and leadership support. Yet, no single method guarantees success – in fact, research to date highlights that innovations in nursing can take up to 15 years to reach widespread adoption [[4], [5], [6]]. The following two research questions guided this study. 1) What implementation strategies were critical to HRH to ensure integration into daily practice, and 2) What patterns or trends can be identified in the statistical process control charts of the longitudinal data for the delirium measures pre- and post-guideline implementation at HRH.

## 2 Methods

### 2.1 Study design

A retrospective longitudinal evaluation was implemented using statistical process control charts and segmented regression analysis [[7], [8], [9]]. Delirium-specific process and outcome evaluation indicators were obtained to assess the effect of BPG Implementation. The process and outcome indicators were plotted on run charts, which were used to calculate the Central Line, Upper Control Limit (UCL), and Lower Control Limit (LCL) to evaluate event spikes. Segmented regression analyses were used to determine critical points following the BPSO® designation and sustainment periods.

### 2.2 Ethical considerations

This project was undertaken as a quality improvement initiative at HRH, in compliance with the hospital policy entitled “Quality Improvement/Quality Assurance/Research Determination” (Reference #9224, Version number 3). An ARECCI Ethics Screening Tool Report was completed, with a risk score of 0, and submitted to the Research Ethics Office. All procedures comply with both institutional policies and Tri-Council Policy Statement-2 (2022) Article 2.5 for quality improvement and program evaluation activities.

### 2.3 Identification and analysis of problems

In reviewing the most prominent hospital-associated risks related to everyday practice at HRH, the nursing executive leadership implements the RNAO BPG relevant to delirium, dementia & depression in Older Adults, as older adults comprise approximately 71 %–73 % of HRH’s population. In particular, delirium has associations with predicting patient morbidity, increased hospital length of stay, and is considered preventable. Delirium is believed to be caused by a complex combination of predisposing factors (e.g., frailty, cognitive impairment) and precipitating factors (e.g., surgery, immobility, noise), underscoring the multiple mechanisms underlying delirium. Moreover, the BPG recommends non-pharmacological prevention and intervention for at-risk older adults to treat delirium. Thus, to prevent and treat delirium, HRH needed to adopt appropriate screening measures. A baseline gap analysis was completed for delirium screening, revealing variable practices at an individual patient-clinician level to the organizational level. Some contributory factors identified included documentation burden, non-standardized prompts, and limited situational awareness at the unit/organizational levels. These gaps informed a multifaceted implementation strategy targeting documentation, education/champion development, and real-time alerting.

### 2.4 Nursing Quality Indicators for Reporting and Evaluation (NQuIRE) reporting and compliance

As part of the RNAO BPSO® Program, organizations regularly report systematic monitoring of process and outcome indicators for each implemented BPG to the RNAO through the NQuIRE database. NQuIRE is critical to the Knowledge-To-Action Framework, as it provides organizations with data measurement, feedback, and benchmarking infrastructure. In fact, real-time reporting infrastructures created at HRH improved the rapid identification of gaps in practice, supported evidence-based decision-making, and ensured the enhanced implementation of interventions such as the Cognition-Hydration-Agitation-Sleep-Mobility (CHASM) care bundle [1]. Of note, the HRH nursing leaders regularly reported these indicators to the hospital’s Board Quality Assurance Committee, aligning with the Excellent Care For All Act (2010) legislative requirement for hospitals to stay up to date. Given the traditional difficulties of translating research into practice, high-reliability organizations should translate evidence-based practice into routine practice as much as possible [10,11] to minimize and eliminate practice gaps. As research continues to develop robust, universally applicable methods for guideline translation [[10], [11], [12], [13]].

### 2.5 Formation of local characteristics guidelines

#### 2.5.1 Description of guidelines from the Registered Nurses' Association of Ontario

The RNAO Delirium, Dementia, and Depression Guideline provides 16 recommendations that are general and/or specific to delirium. We added the Short Confusion Assessment Method (CAM) [1] and CHASM interventions [1] to all relevant delirium assessment and intervention screens. These changes prompted staff to follow the required practices outlined in the BPG to help prevent delirium.

#### 2.5.2 Establishment of an improvement team

HRH established an interprofessional, cross-functional improvement team of approximately 30 clinical leaders to implement all 16 recommendations and good practice statements (Appendix A). The majority of the group consisted of clinical practice leaders, managers, and directors from various programs. Leaders were selected from units with patient demographics of interest (e.g., patient encounter aged 65 or older) and excluded the pediatric unit and the birthing units. All team members completed the gap analysis and began adapting recommendations to the local unit’s practices (e.g., medicine versus surgery). The organization BPSO® led, the Directors of Professional Practice, and the Director of Quality & Patient Safety were responsible for creating Command Center tile algorithms with GE Healthcare Partners. The clinical practice leaders were then tasked with implementing the teaching component by developing orientation and screening tools within the electronic medical record (EMR) to be used upon admission to the hospital.

Another key stakeholder group in the implementation of the BPG was our frontline staff. Following the implementation of screening tools within the test domain of the EMR, a group of 30 nurses, primarily from the Nursing Resource Team (NRT), was directly involved in user acceptability testing. NRT nurses are cross-trained across multiple departments and have a broader skill set and experience across multiple patient populations. User acceptability testing was critical for the frontline staff to leverage their experience and provide feedback on the newly developed delirium screening tool.

#### 2.5.3 Localized improvement

Using three different implementation strategies, we translated the delirium-specific BPG and evidence-based practice decision-making into HRH-specific workflows, with minimal modifications, as the recommendations were easily adopted. We describe the strategies in more detail in the subsequent sections. 1) EMR “DocOp” (2017–2022): Embedding BPG parameters for standardized screens; added forcing functions for required text fields and time-based work/task prompts (“clock icons”); aligned fields for future data extraction (NQuIRE and Command Center). 2) Education and BPG Champions (2017–2022): Redesigned orientation using adult learning principles (i.e., Knowles; Kolb; flipped classroom), embedded delirium content in simulations, and concurrently expanded the Best Practice Champion model to all new hires and existing staff. 3) Command Center Integration (2018–2019): Built a BPSO Risk of Harm tile incorporating algorithm triggers for the BPG of interest; designed stratified alerts by role and urgency; defined expected response times and escalation thresholds.

##### 2.5.3.1 Strategy 1 - Documentation optimization (DocOpt) at HRH (2017–2022)

To achieve high reliability, HRH leadership launched DocOpt to standardize documentation and embed RNAO BPG principles, ensuring the renewed EMR would drive nursing practice excellence. Multiple cross-functional teams analyzed clinical workflows by reviewing EMR screens for evidence-based content, integration of BPGs, which interventions needed to be surfaced, whether relevant information was pertinent to the patient’s story, and whether it met regulatory or reporting requirements. As the goal was to achieve optimal efficiency for nursing documentation, ensuring all screens were thoroughly reviewed spanned five years. Reporting requirements were also taken into account, including the anticipated reporting of BPG metrics to NQuIRE. The guiding principles for the HRH DocOpt project are in Appendix B.

Using an interoperability design principle, the hospital prioritized data availability to all relevant digital systems to facilitate critical communication. For example, if a patient were assessed in-the-moment as high falls risk, the documentation in the EMR would trigger patient flags in various systems, including status boards at the nursing station and room-side monitors. The DocOpt project identified adding “clock icons” to new nursing tasks in the EMR as a direct reminder to staff to ensure timely completion and to improve situational awareness (e.g., patient status boards, Command Center) [[13], [14], [15]]. Wherever possible, HRH planned for forcing-function elements [[16], [17], [18]] in the EMR to ensure mandatory fields were completed, thereby flagging missed care tasks. Regarding delirium, CAM and CHASM assessments were embedded in the EMR. Data reports were created so it would be easy to pull electronically in anticipation of reporting the data administratively to HRH leaders and RNAO’s NQuIRE portal.

##### 2.5.3.2 Strategy 2 - Orientation enhancements and best practice champion model (2017–2022)

HRH nursing leadership reimagined staff orientation to align with high-reliability and BPSO principles. The eight-day curriculum shifted from didactic delivery to experiential-based learning, incorporating Knowles’ Andragogy Model [19], Kolb’s Experiential Learning [20], the Flipped Classroom Principles [21], and Tanner’s Clinical Judgment Model [22]. We incorporated case study simulations and storytelling within the orientation to encourage learners to interact with the content, as proposed by the aforementioned models.

The two-year orientation redesign integrated RNAO BPGs into modules, materials, and simulations. A new handbook provided pre-orientation content, so in-class time focused on clinical problem-solving. Protocols linked to BPGs were clearly identified, and expected clinical practices were laid out for staff. Interactive delirium training sessions were added to staff orientation. The training session covered recognizing delirium in patients, screening with CAM, what to do when patients are flagged as CAM-positive, and treating delirium after onset. The training also featured case studies so that staff would apply their new knowledge and interact with the curriculum content. Similar refresher training was also provided to existing staff through annual skills days. Grinspun et al. [3] highlight best practice champions as key to spreading evidence-based care. Cox et al. [23] also emphasize the importance of champions and their role in educating, mentoring, and influencing practice leaders. Thus, HRH set new expectations for all new hires and existing staff to become BPG champions, ensuring uniform translation of best practices into unit contexts.

##### 2.5.3.3 Strategy 3 command center tile creation (2018–2019)

HRH leveraged its EMR and digital infrastructure to advance patient safety and quality through the Command Center—a 24/7 hub with 33 real-time data tiles. Data from the EMR and other systems feed into the predictive engine, which analyzes up to 120,000 data points per minute using machine learning to flag patients at risk. The algorithm design took into account the Heuristic Principles as described by Zhang et al. [24], the Work Domain Analysis Framework [25], and the Principles of Evidence-Based Practice for Safety [26]. Appendix C shows a more detailed description of the HRH Delirium algorithm. Alerts are prioritized by urgency and linked to clear response timelines; icons shift colors if unresolved. Each tile displays patient details and triggers based on RNAO BPG indicators, supporting timely interventions. For delirium, the command center tile would flag if a patient had a positive CAM score but had not yet received a delirium CHASM protocol from a physician’s electronic order within 4 h. Appendix C provides a more detailed description of the HRH Delirium algorithm.

### 2.6 Application of the registered nurses’ association of ontario best practice guidelines

The Knowledge-to-Action Framework [27] was reviewed in consideration of the overall design and delivery of the various implementation activities undertaken at HRH. The journey for HRH in following the steps of the Knowledge-to-Action Framework began with conducting a gap assessment, identifying the corresponding RNAO BPG, and planning for implementation and sustainment activities. The three key implementation strategies (described previously; sections 2.4.3.1-2.4.3.3) were developed, staged and executed) and reported in accordance with the Standards for Reporting Implementation Studies: the STaRI checklist for completion [28].

#### 2.6.1 The setting

HRH is a multi-site, community-based acute care hospital in Toronto, serving a catchment of approximately 850,000 individuals. HRH discharged an average of 28,167 patients annually, ranging from 23,149 (in 2014/15) to 33,073 (in 2024/25). The study population comprised all hospitalized adults with quarterly aggregation at the organizational level.

#### 2.6.2 Data collection, including evaluation indicators and collection method

Administrative data was extracted quarterly from the EMR for the RNAO NQuIRE reporting and local quality dashboards (de-identified and aggregated). For the delirium BPG, a process and outcome indicator was used for evaluation. The process indicator selected at HRH for process compliance was the percentage of older adults assessed for delirium risk factors upon first contact at admission (i.e. number of older adults assessed for delirium/total number of older adults admitted at HRH) [1]. The outcome indicator selected at HRH for patient delirium was the number of delirium occurrences/total number of patient days (i.e. delirium occurrence per 1,000 patient days) [1].

In addition, although patient satisfaction is not a formal requirement for NQuIRE reporting, all hospitals in Ontario conduct and publicly report patient satisfaction scores. These scores were considered in anticipation of improved patient outcomes resulting from the implementation of the BPG. The patient satisfaction surveys cover a wide range of topics, specifically inquiring about the patient’s satisfaction regarding their care from nurses and physicians, the hospital environment, their experience at admission/discharge, and their overall thoughts, using a combination of Likert scales and open-ended questions.

#### 2.6.3 Data analysis

All quarterly administrative data were plotted as run charts in Microsoft Excel to identify shifts and runs initially. We then constructed Statistical Process Control (SPC) charts in Microsoft Excel Macros, estimating centrelines and upper/lower control limits for each process and outcome indicator. We examined special-cause signals (e.g., runs and shifts beyond control limits). Segmented regression analysis of the observed data (rate of delirium onset per 1,000 patient days) was performed in Microsoft Excel Macros for sensitivity testing of the variables pre- and post-designation. This analysis identifies trends pre- and post-implementation, as well as any transition period(s) the organization experienced [[7], [8], [9]].

## 3 Results

### 3.1 DocOpt program

Overall, the DocOpt project reduced the HRH EMR from 646 assessments of.

396 screens, 80 duplicates were identified and archived. Nursing staff provided anecdotal feedback on improved assessment completion and compliance, screen “usability”. All BPG-specific data points were confirmed for data flow to the HRH Command Center. The estimated project cost for DocOpt was $1.6M, and it was completed 30 % under budget.

### 3.2 Process and outcome indicators

As a result of implementing the delirium-specific recommendations, there has been an observed shift and 1 % improvement in the process measure for delirium (i.e. the percentage of older adults assessed for delirium/total number of older adults admitted at HRH) over six years, but a corresponding shift and 1.4 (23.3 %) median decrease in delirium occurrence in older persons per 1,000 patient care days (Appendix D and Appendix E). With respect to our process indicators shown in Appendix F, the Center line $\left(\bar{p}\right)$ was calculated as 93.32 %, the Upper Control Limit (UCL) ≈ 100.81 %, and the Lower Control Limit (LCL) ≈ 85.84 % *(with the n = 100 assumption).* The signals that were identified for this chart include: 2021-2022 Q1 (85.4 %) – below LCL (special cause), 2021-2022 Q2 (82.7 %) – below LCL (special cause). No quarters were above UCL (as expected with a high compliance process).

Conversely, for our outcome indicator of delirium occurrences per 1,000 patient days in Appendix G, the Center line $\left(\bar{X}\right)$ was calculated for = 5.236/1,000 PD, and estimated $\hat{\sigma }$ = 1.197 (from MR). The Upper Control Limit (UCL) = 8.826 and Lower Control Limit (LCL) = 1.646. The signals determined for this outcome measure are 2020-2021 Q1 (11.6) – above UCL (special cause). After the 2020-2021 Q1 spike, the outcome series returns to and remains within its limits, with gradual stabilization around the mean in 2022–2025 (Appendix G). Segmented regression for Delirium Cases per 1,000 Patient Care Days (Outcome) at HRH from 2018 to 2025 was analyzed using April 1, 2021, as the breakpoint (Appendix H). The R-squared calculated for this segmented regression is: 0.141 (low explanatory power). Coefficients include time: −0.0568 (not significant), post-implementation: −0.5907 (slight level decrease, not significant), time after: −0.0058 (virtually no slope change), and percentage Screened: +0.0125 (minimal effect). Moreover, a transitional period was observed between Q3 of 2019/2020 and Q1 of 2021/2022, encompassing the update of documentation screens and the launch of the Command Center BPSO® Tile (Appendix H).

### 3.3 Best practice champions and patient satisfaction

RNAO requires all BPSOs® to train at least 15 % of staff as best-practice champions. Instead of 300 staff, HRH has certified 1,116 best-practice champions to date. Best-practice champions also provided feedback on the impacts on nursing workload and workflows. Post-user acceptability testing, 171 nurses reported that 83 % believed DocOpt would benefit their clinical practice by reducing time spent on documentation and increasing time available to provide patient care. Additionally, 90 % of the nurses reported that they found it easy to integrate the revised screens into their current clinical workflows. However, 100 % of the nursing staff reported concerns about the project schedules, noting that additional time for planning the rollout would have been appreciated.

Given the clinical significance of the process and outcome measures, the BPG, we also noted a minor shift in patient satisfaction scores. In 2018, HRH received a topbox score of 88.9 % on 8,232 surveys, indicating that patients would *definitely* recommend HRH to their friends and families for care. In 2025, this topbox score increased slightly to 90 % (from 3,721 surveys) of patients would *definitely* recommend this hospital to their friends and family; though this score is an incremental increase, it was not found to be statistically significant (*P* = 0.086).

## 4 Discussion

HRH saw a reduction in delirium cases per 1,000 patients during the BPSO sustainment period. While the run chart for screening percentage indicated only a 1 % improvement in patient CAM screening, the resultant 23.3 % decrease (1.4 median decrease) in delirium cases may be attributed to multiple factors. The two key strategies that have had the greatest influence are the revised delirium EMR screen (which was redesigned to align with the CAM and CHASM interventions) and flagging patients in our Command Center who had a CHASM protocol overdue. The documentation quality to drive nursing practice and interoperability with the Command Center, as a backstop for safety, was pragmatic and efficient.

In addition, the large number of best-practice champion training sessions at HRH was important to ensure that the majority of staff were responsive to patient delirium. Close and continuous monitoring of real-time reporting not only supports better clinical practice but also ensures accurate reporting of the measures used by HRH and the NQuIRE portal. Ultimately, this rapid feedback loop presented the opportunity to reduce the research-to-practice lag at HRH. Training was developed to be engaging and interactive, and featured simulations surrounding patient delirium.

With respect to process and outcome measures, SPC charts provided valuable insights into process stability and variation over time – we were able to identify a few special-cause variation points, which, in turn, were explainable. The SPC charts set the stage for sensitivity testing, and segmented regression analyses allowed for examining relationships wherever possible, pre- and post-BPSO designation, as the context in which nursing practice was occurring at HRH. However, an additional contextual factor to note is the uncertainty surrounding the COVID-19 pandemic and various factors impacting safe care delivery across all hospitals, as CIHI noted a Canada-wide trend of increasing patient harm during the pandemic [29]. The HRH experience aligns with current literature in that healthcare systems are experiencing a new normal of increased patient illness severity, as there appears to be significantly higher patient acuity in the first quarter of 2024 compared to the fourth quarter of 2019 [30,31].

Regarding delirium, run charts indicated that, despite initial variability, stability is achieved for both process and outcome measures at HRH. For our process indicators, leadership confirmed that the hospital experienced shifts in the nursing workforce post-pandemic, which led to the two detected signals. Similarly, with respect to the outcome indicator, the spike was likely related to the increase in COVID-19 cases as the World Health Organization declared the pandemic on March 20, 2020. The importance of improving the quality of delirium screening (by adding the short CAM and CHASM interventions) was a key EMR element for further developing digital clinical decision-making and risk-stratification tools [32]. As EMRs have already demonstrated potential for rapid communication between clinicians and service teams, the real-time documentation of interventions also serves patient flow purposes well, and hospitals optimize their EMRs to serve multiple strategies in addition to improving patient safety [15,16,33,34].

The Knowledge-to-Action Framework [27] provided an excellent implementation framework to work through adaptation at the local HRH context, including standardizing implementation of CAM and CHASM with respect to the utilization and reporting for real-time data [27], as well as the utility of Command Centers that immediately improve situational awareness for the staff as well as backstops for safety [35]. In alignment with the current literature [3,36,37], both frontline staff and leadership engagement were critical success factors for HRH implementation of the BPGs, especially in the creation and implementation of the Command Center BPSO® Tile. Meaningful triggers were required for successful stratification of alerts and their respective corrective actions; stratified alerts also support data-driven decision making without replacing clinical judgment [38,39]. Additionally, direct support came from HRH senior management, as Matthew-Maich et al. [36] highlight, through widespread engagement, multi-pronged strategies can then be planned for maximum impact at all levels of an organization.

Based on the reporting requirements for process and outcome measures, HRH has sustained BPG implementation [37,40]. However, planning for a single data point (such as an assessment, intervention, or patient outcomes) to serve multiple purposes demonstrates true optimization of EMR. Taneva et al. [14] noted that a singular assessment data not only serves as information of care provided, but also a demonstration of nursing performance and its sustainment, and also an opportunity for flagging potential safety risks for escalation, and potentially a data point for research on nursing practice and patient outcomes [14,37]. Though clinical practice guidelines are not to be confused with algorithm-based clinical decision tools [38], this team suggests that translating the BPG into Command Center tiles was successful across all levels of nursing care in a meaningful way. The BPG provided the HRH team with triggers to consider, which aligned with research literature [38]. In addition, stratified alerts can be constructed to maintain evidence-based practice as a strategic priority and reinforce practice expectations [39]. Custom tiles developed by HRH and GE Healthcare provided clinicians with actionable prompts, including escalation pathways for polypharmacy, pain, and delirium risk. Centralized monitoring enhanced situational awareness, workflow integration, and adherence to best practices. By centralizing data in the Command Center and monitoring indicators, HRH clinicians were empowered to take timely actions to provide safe, high-quality care in alignment with best-practice guidelines. Additionally, sharing NQuIRE data back to the frontline through digital quality dashboards, as well as to the HRH board of directors, demonstrates true top-to-bottom commitment to evidence-based practice, teamwork, and accountability.

Though the implementation team was disappointed that the ideal patient outcomes were not achieved (i.e., complete elimination of hospital-based delirium), we recognize that HRH began its high-reliability journey before implementing the clinical BPGs. As BPG implementation tends to be resource-intensive, leaders need to recognize that increased workload and lack of time require creative thinking and opportunities to explore, implement, and evaluate change ideas [40,41]. The value of implementing, evaluating, and sustaining BPG will always have high clinical value [37], with the potential to improve positive institutional outcomes and cultural change [40,41].

The authors note several limitations of this study. The key limitation concerns generalizability, as context-specific adaptation is a significant component of the Knowledge-to-Action Framework [27,42]. As a single-institution evaluation with system-level changes, attribution to any single component is constrained. This comprehensive approach at HRH used a Command Center; however, very few organizations have one available. Next, we recognize the limitation regarding the resource intensity of the BPG implementation. The authors also recognize that another study may be required in years to come, as traditional research shows a delay of 15 to 20 years for innovative practices to be fully embedded in daily work, along with improved situational awareness and the corresponding organizational support.

Regarding data, quarterly administrative data was analyzed to assess process adherence. Quarterly aggregation of data may, in fact, mask short-term fluctuations and certain seasonal patterns that are more apparent on a daily or weekly basis (e.g., as hospitals operate on day and night shifts and on weekday and weekend schedules). The large aggregation of data may also retrospectively reduce the number of time points available for analysis, thereby reducing statistical power to detect leveling or slope changes in the interrupted time series and, consequently, impacting and delaying feedback mechanisms as part of the Knowledge-To-Action Framework.

Although a high-level overview of the initiatives has been provided in this paper, the authors acknowledge that no patient-level covariates (e.g., demographic data) were included in the analysis, nor was a balance measure regarding actual staff workload. These measures offer opportunities for future research. Though the delirium patient outcomes are promising, we recognize the limitations in generalizing these results, as they require other organizations to exhibit a similar EMR maturity level, interoperability design, and active leadership support. Thus, our results should be interpreted within the context of quality improvement rather than randomized efficacy studies.

## 5 Conclusion

Real-time data and long-term evaluation of BPG metrics within a highly structured BPSO® program help organizations gauge and explore their BPG implementation within an organizational context. HRH has seen improvement for patient delirium outcomes in particular. RNAO BPG-based clinical algorithms and stratified triggers and alerts have improved situational awareness for frontline staff, nursing leaders, and Command Center staff to act upon in real-time. SPC analysis confirmed process stabilization at HRH. We attribute our results to the quality of the documentation revisions aligned with the RNAO BPGs.

## Data availability statement

The datasets generated during and/or analyzed during the current study are available from the corresponding author upon reasonable request.

## CRediT authorship contribution statement

**Jennifer Yoon:** Conceptualization, Methodology, Data curation, Formal analysis, Supervision, Project administration, Writing - original draft. **Derek Hutchinson:** Writing-review & editing, Formal analysis, Supervision, Project administration. **Amanpreet Ghuman:** Writing-review & editing, Formal analysis, Supervision, Project administration. **Gurmit Maghera:** Data acquisition, curation, Project administration, Writing - review & editing. **Beatrise Edelstein:** Conceptualization, Writing-review & editing, Project administration. **Rachelle Soogree:** Conceptualization, Project administration, Writing - review & editing. **Maritess Payumo:** Conceptualization, Project administration, Writing - review & editing. **Grace Mercieca:** Conceptualization, Project administration, Writing - review & editing. **Shirley Goguen:** Conceptualization, Project administration, Writing - review & editing. **Elizabeth Leblanc:** Conceptualization, Project administration, Writing - review & editing. **Akhil Plathanathu:** Methodology, Data curation, Formal analysis. **Abhimanyu Sud:** Methodology, Formal analysis, Writing - review & editing. **Barbara E. Collins:** Writing-review & editing, Resources. **Teodora Neagu:** Writing-review & editing. **Aleksandra Zuk:** Supervision, Methodology, Writing - review & editing.

## Declaration of competing interest

The authors have declared no conflict of interest.
