# Supplementary material for: Implementation of the Registered Nurses’ Association of Ontario Best Practice Guidelines for delirium-specific recommendations in a digital practice setting at Humber River Health, Canada
Source: Int J Nurs Sci. 2026 Apr 25;13(3):323–9. doi: 10.1016/j.ijnss.2026.04.015 (PMC13245496; doi:10.1016/j.ijnss.2026.04.015)
Supplement: Multimedia component 1 [file mmc1.docx]

Appendix A Recommendations and good practice statements

| Phase | Recommendation |
| --- | --- |
| 1.1 | Establish therapeutic relationships and provide culturally sensitive person- and family-centred care when caring for and providing education to people with delirium, dementia, and depression and their families and care partners. |
| 1.2 | Identify and differentiate among signs and symptoms of delirium, dementia, and/or depression during assessments, observations, and interactions with older persons, paying close attention to concerns about changes expressed by the person, his/her family/care partners, and the interprofessional team. |
| 1.3 | Refer older adults suspected of delirium, dementia, and/or depression to the appropriate clinicians, teams, or services for further assessment, diagnosis, and/or follow-up care. |
| 1.4a | Assess the person’s ability to understand and appreciate information relevant to making decisions and, if concerns arise regarding the person’s mental capacity, collaborate with other members of the health-care team as necessary. |
| 1.4b | Support the older person’s ability to make decisions in full or in part. If the older person is incapable of making certain decisions, engage the appropriate substitute decision-maker in decision-making, consent, and care planning. |
| 1.5 | Exercise caution in prescribing and administering medication to older adults (within the health-care provider’s scope of practice), and diligently monitor and document medication use and effects, paying particular attention to medications with increased risk for older adults and polypharmacy. |
| 1.6 | Use principles of least restraint/restraint as a last resort when caring for older adults. |
| 2.1 | Assess older adults for delirium risk factors on initial contact and if there is a change in the person’s condition. |
| 3.1 | Develop a tailored, non-pharmacological, multi-component delirium prevention plan for persons at risk for delirium in collaboration with the person, his/her family/care partners, and the interprofessional team. |
| 4.1 | Implement the delirium prevention plan in collaboration with the person, his/her family/care partners, and the interprofessional team. |
| 4.2 | Use clinical assessments and validated tools to assess older adults at risk for delirium at least daily (where appropriate) and whenever changes in the person’s cognitive function, perception, physical function, or social behaviour are observed or reported. |
| 4.3 | Continue to employ prevention strategies when caring for older adults at risk for delirium who have not been identified as having delirium. |
| 4.4 | For older adults whose assessments indicate delirium, identify the underlying causes and contributing factors using clinical assessments and collaboration with the interprofessional team. |
| 4.5 | Implement tailored, multi-component interventions to actively manage the person’s delirium in collaboration with the person, the person’s family/care partners, and the interprofessional team (level of evidence = Ia).  These interventions should include:  • treatment of the underlying causes (level of evidence = Ia),  • non-pharmacological interventions (level of evidence = V), and  • appropriate use of medications to alleviate the symptoms of delirium and/or manage pain |
| 4.6 | Educate persons who are at risk for or are experiencing delirium and their families/care partners about delirium prevention and care. |
| 5.1 | Monitor older adults who are experiencing delirium for changes in symptoms at least daily using clinical assessments/observations and validated tools, and document the effectiveness of interventions. |

Appendix B Guiding principles for the HRH DocOpt project

| Aspects | Measures |
| --- | --- |
| Driving high reliability | Drive standardized practices and processes through  documentation |
| Prioritizing safety | Identify and fix current documentation that pose a risk to patient safety |
| Ensuring standardization | Standardization of documents in accordance to the RNAO clinical practice guidelines |
| Ensuring efficiency | Streamlining and reducing duplication of documentation as different screens were identified to have similar assessment fields |
| Improving quality | Improve the quality of the EMR screens by incorporating best practice guidelines and quality based procedures into HRH clinical documentation |

Appendix C. The HRH-designed Delirium Algorithm for the BPSO Tile


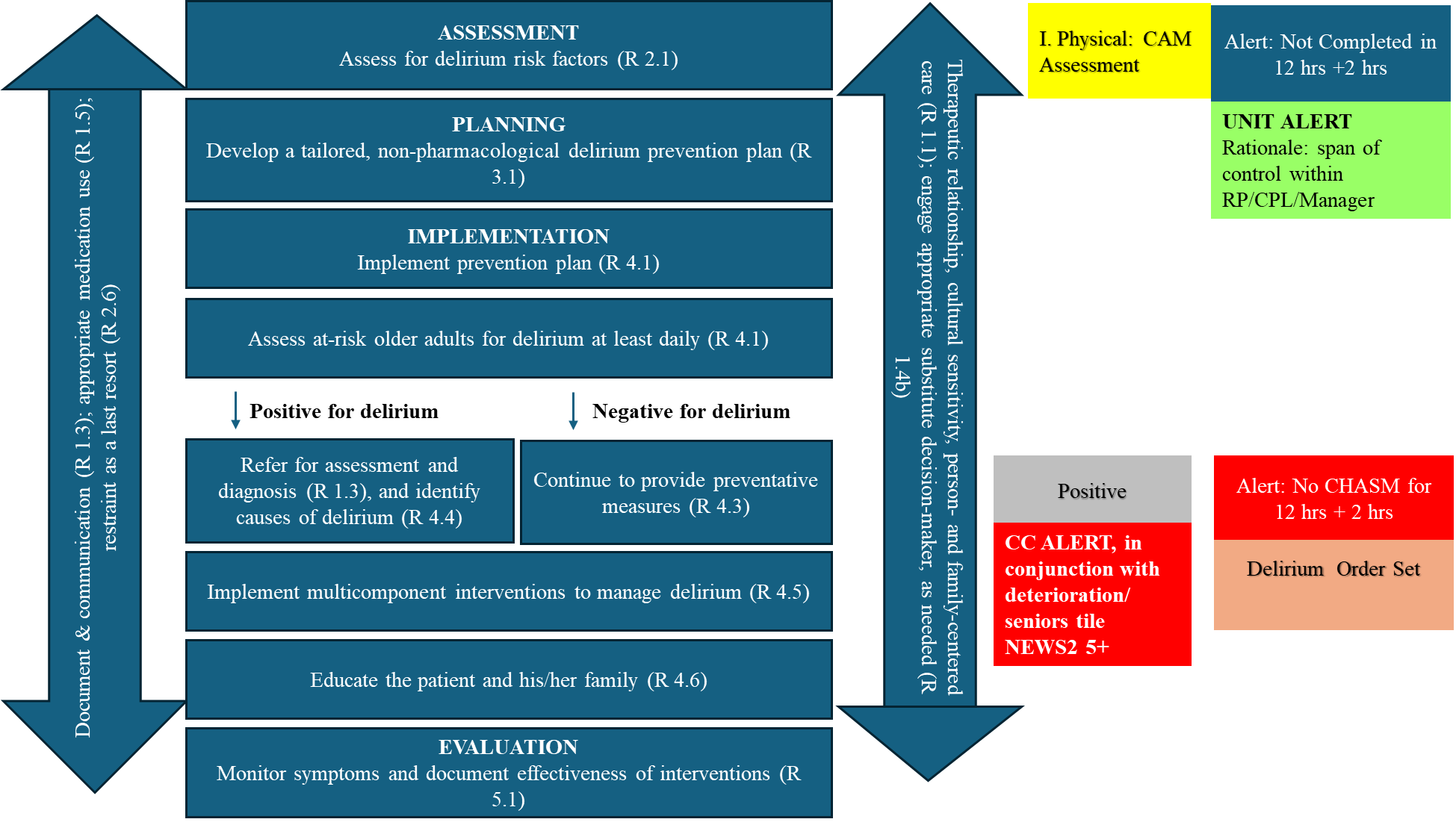


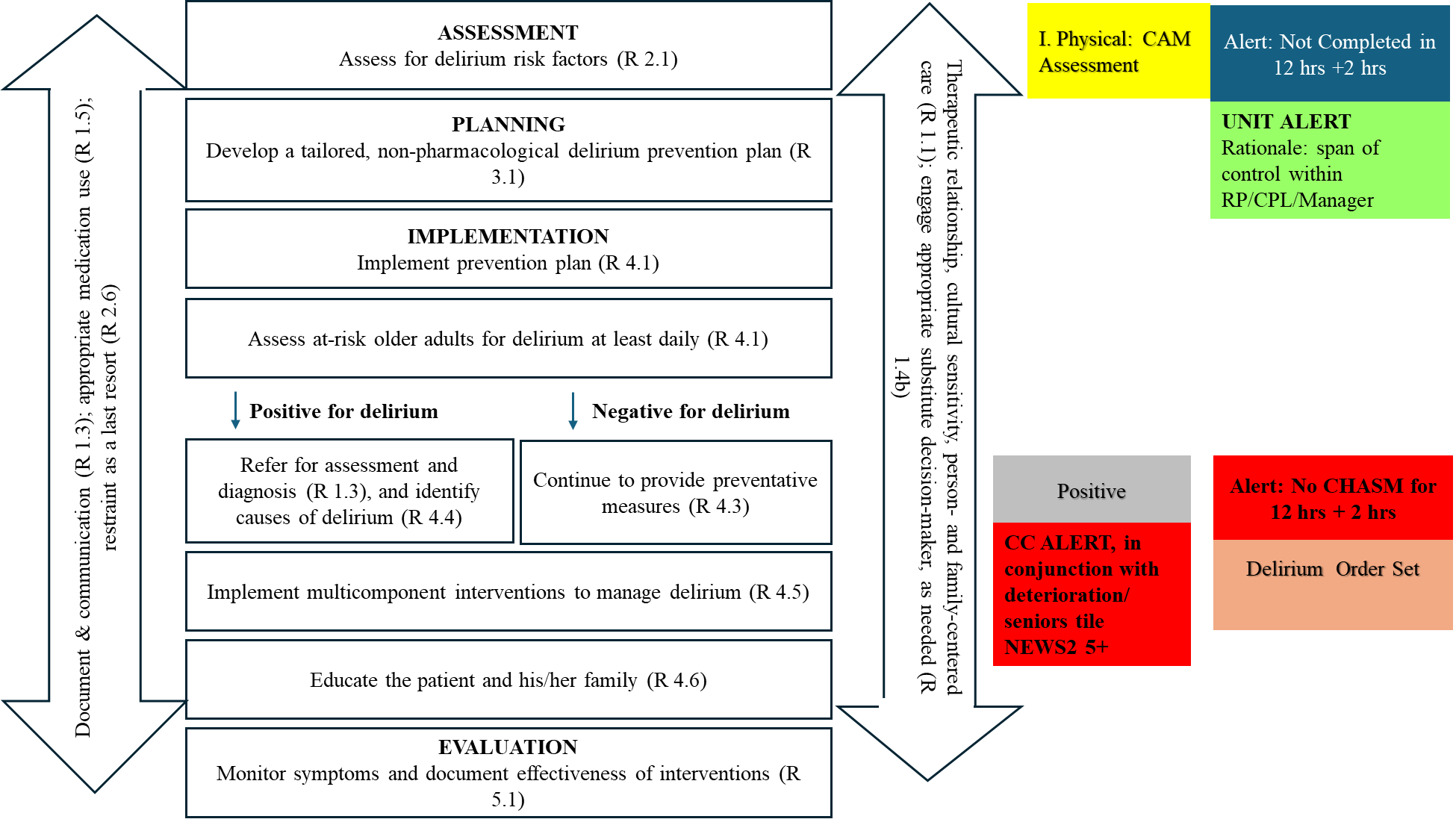


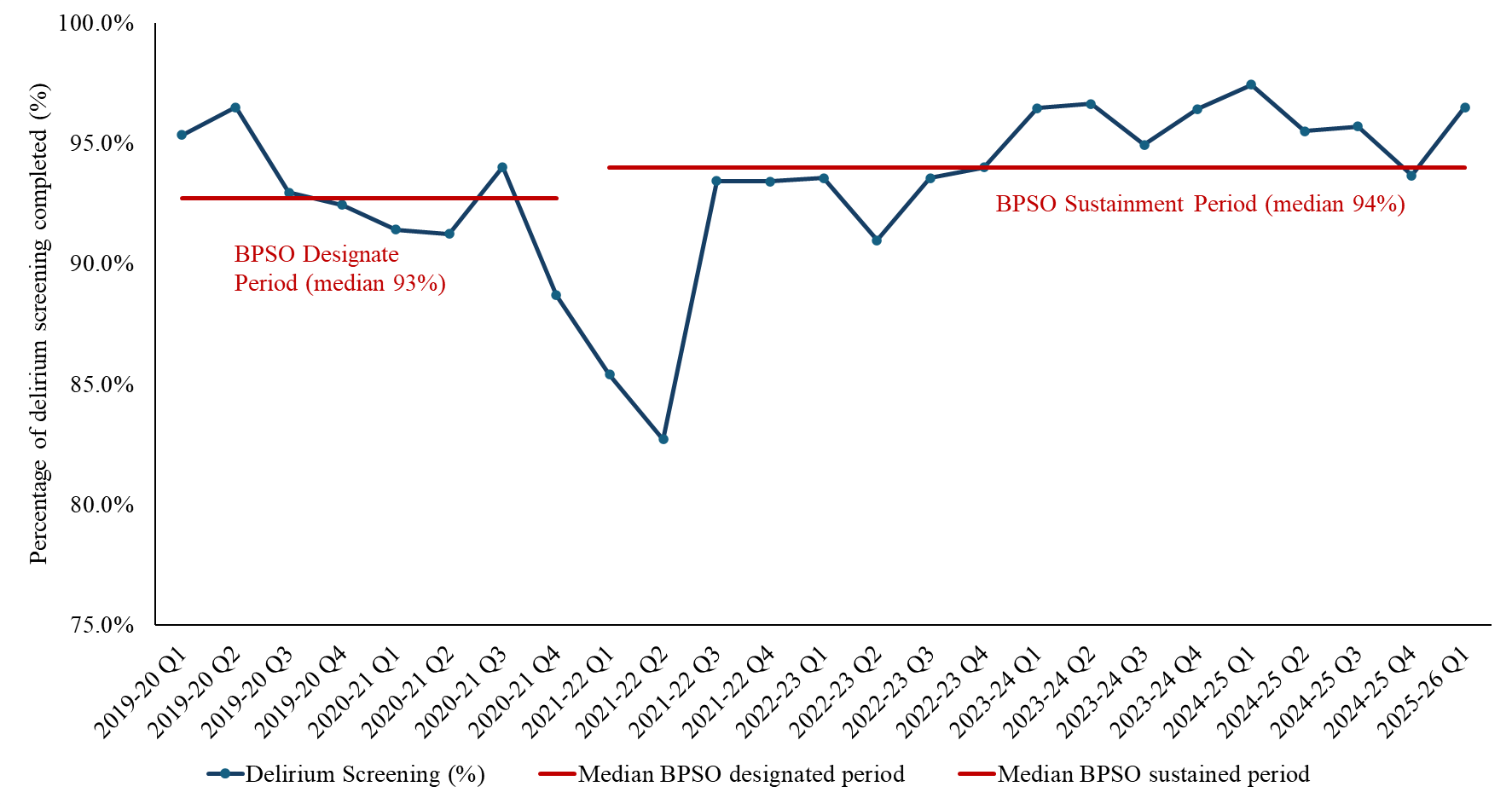


**Appendix D.** Process indicator run chart for reported delirium assessment measures at HRH from 2019-2025, reported as a percentage of patient screening completed. Q = Quarter. BPSO = Best Practice Spotlight Organization.


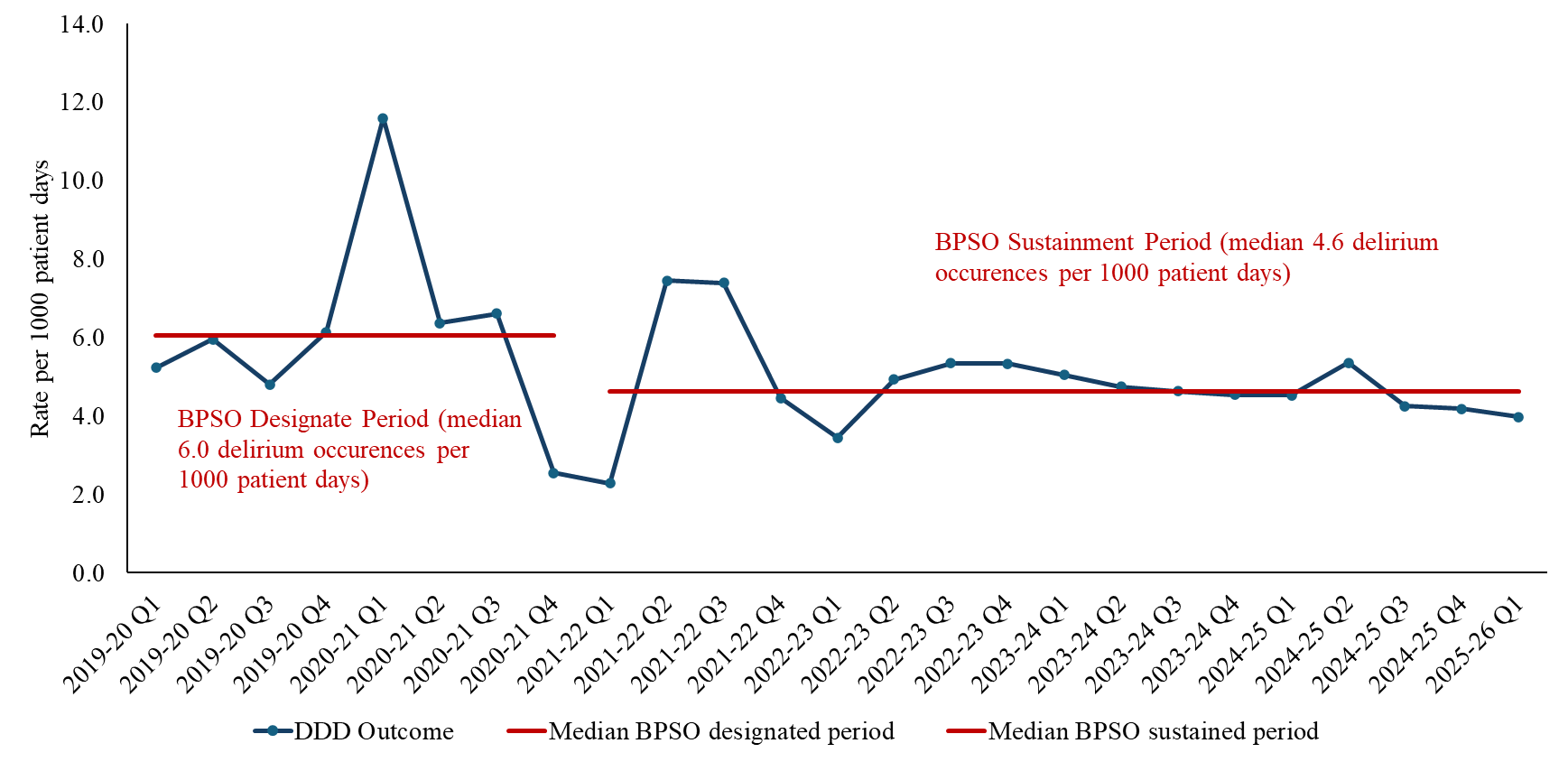


**Appendix E.** Outcome indicator run chart for reported delirium cases per 1,000 patient care days (outcome) at HRH from 2019-2025. Q = Quarter. BPSO = Best Practice Spotlight Organization.

**
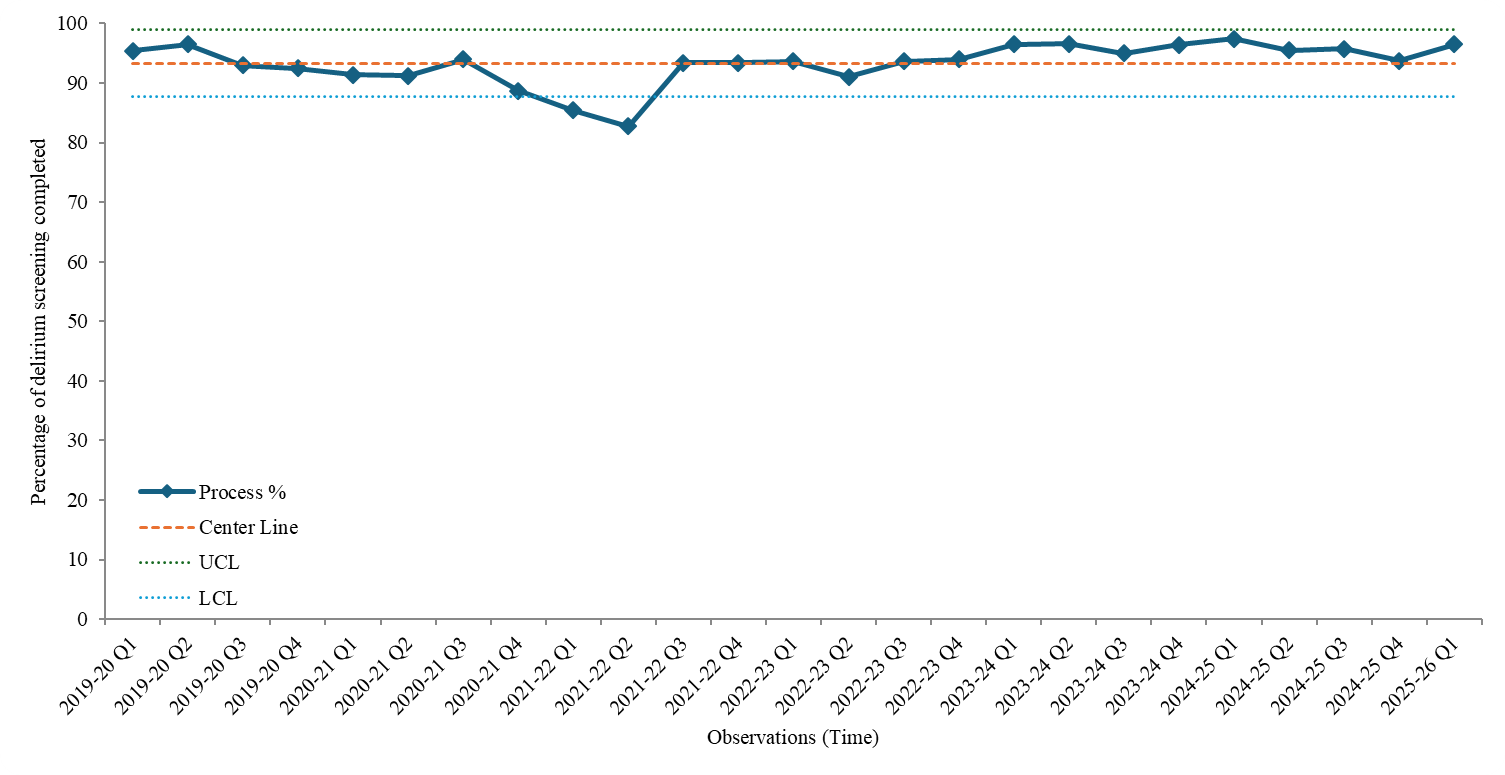
**

(%)

**Appendix F.** Statistical Process Control Chart for Delirium Assessment for process indicators at HRH from 2019-2025. Q = Quarter. UCL = Upper Control Limit. LCL = Lower Control Limit

**Appendix G.** Statistical Process Control Chart for Delirium Cases per 1,000 Patient Care Days for outcome indicators at HRH from 2019-2025. Q = Quarter, UCL = Upper Control Limit, LCL = Lower Control Limit


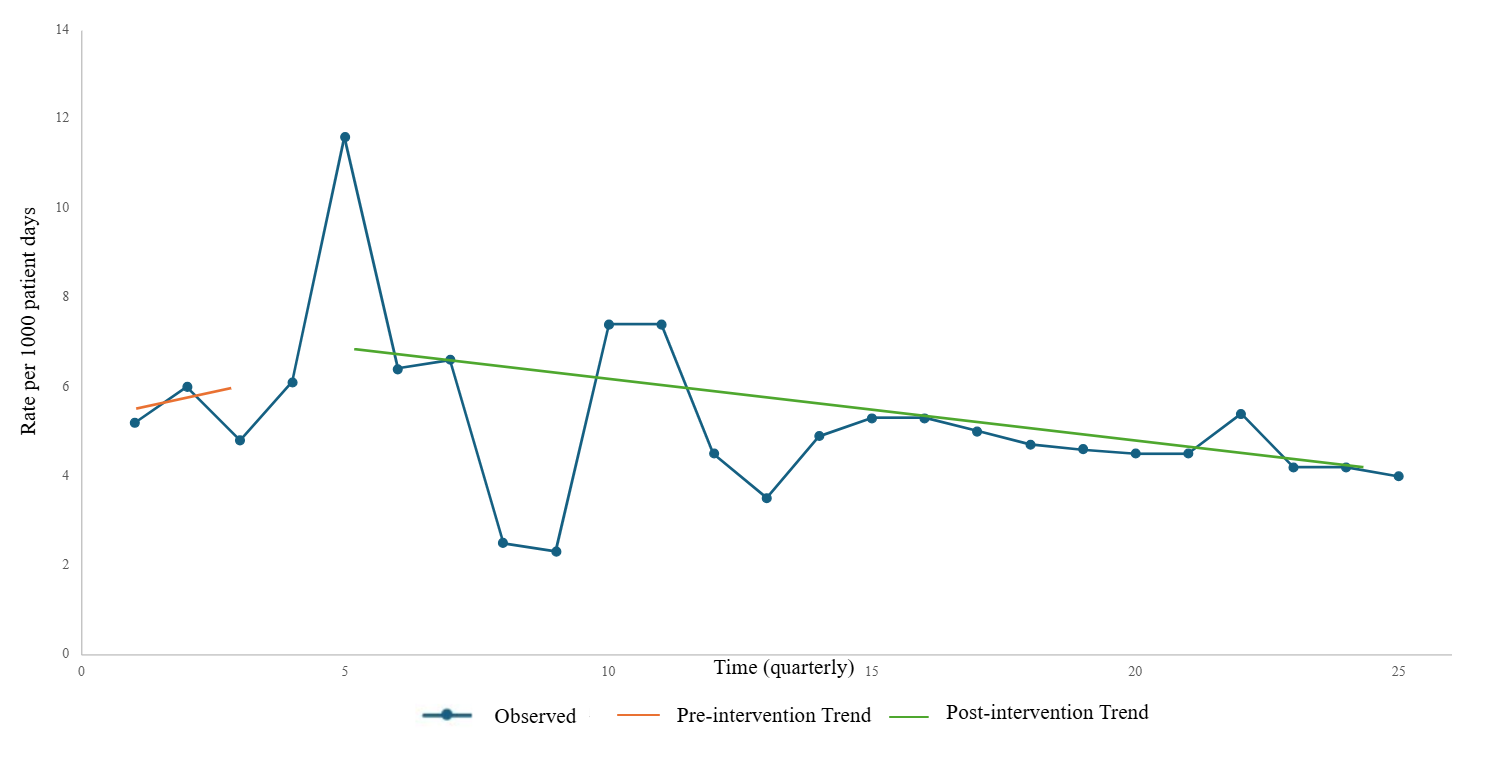


**Appendix H.** Segmented regression for Delirium Cases per 1,000 Patient Care Days of outcome indicators at HRH from 2018-2025 analysis using April 1, 2021 as the breakpoint.
