# Supplementary material for: Implementation of the Registered Nurses’ Association of Ontario Best Practice Guidelines for delirium-specific recommendations in a digital practice setting at Humber River Health, Canada
Source: Int J Nurs Sci. 2026 Apr 25;13(3):323–9. doi: 10.1016/j.ijnss.2026.04.015 (PMC13245496; doi:10.1016/j.ijnss.2026.04.015)
Supplement: Multimedia component 2 [file mmc2.docx]

数字化医疗环境下谵妄最佳实践指南的临床实施效果

Jennifer Yoon, Derek Hutchinson, Amanpreet Ghuman, Gurmit Maghera, Beatrise Edelstein, Rachelle Soogree, Maritess Payumo, Grace Mercieca, Shirley Goguen, Elizabeth Leblanc, Akhil Plathanathu, Abhimanyu Sud, Barbara E. Collins, Teodora Neagu,Aleksandra Zuk

【**摘要**】

**目的** 该研究描述了汉伯河健康医疗中心（Humber River Health, HRH）护理管理层，为推动谵妄、痴呆及抑郁相关最佳实践指南持续落地实施的核心策略并探讨其效果。

**方法** 2017年，HRH成为安大略省注册护士协会认证的最佳实践组织，并实施谵妄最佳实践指南。医院在实施指南的基础上，还同时采用电子病历“DocOpt”、将谵妄内容嵌入员工培训、构建健康风险图块并进行分层预警3项主要措施。于2019年至2025年，采用过程指标、结果指标及患者满意度对医院每年入院患者进行回顾性纵向评估。通过 Microsoft Excel QI 基础软件中的统计过程控制图表对过程和结果指标进行了评估，计算中心线、上线及下线。此外，还进行分段回归分析，以评估最佳实践指南实施过程中的关键时间段。

**结果** 从2019年至2025年HRH患者谵妄的过程指标有所改善，且每 1 000 例患者护理日中老年人谵妄的发生率相应下降，减少了 1.4 次（占 23.3%）；90%以上的患者及家属对医院护理质量满意。根据分段回归分析，表明2019年第3季度至2020年第3季度，2021年第1季度至2022年第1季度之间存在过渡期，这标志着应用“DocOpt”系统和健康风险图块时期。

**结论** 在高度结构化的最佳实践组织项目中，实时获取最佳实践指南指标数据及对其进行长期评估，有助于在组织环境中评估和探索指南实施情况。未来研究可依托常态化动态数据监测体系，为最佳实践指南的本土化适配、常态化推行及质量持续改进提供更多实证依据。

【**关键词**】最佳实践指南；谵妄；电子健康记录；循证实践；护理；患者安全

通信作者：Jennifer Yoon, E-mail:[jyoon@hrh.ca](mailto:jyoon@hrh.ca)
